# The potential of dibenzazepine carboxamides in cancer therapy

**DOI:** 10.3389/fphar.2025.1564911

**Published:** 2025-03-28

**Authors:** Nastaran Afsordeh, Safura Pournajaf, Javad Mirnajafi-Zadeh, Mohammad Hossein Pourgholami

**Affiliations:** Department of Medical Physiology, Faculty of Medical Sciences, Tarbiat Modares University, Tehran, Iran

**Keywords:** dibenzazepines, carbamazepine, oxcarbazepine, eslicarbazepine, cancer therapy

## Abstract

Cancer is a leading cause of mortality worldwide, with most conventional treatments lacking efficacy and having significant challenges like drug resistance. Finding new molecules is quite challenging in terms of cost, time and setbacks. Hence, drug repurposing is considered sensible for skipping the long process of drug development. Dibenzazepine carboxamides, as traditional anticonvulsants, primarily function by blocking voltage-gated sodium channels, which not only mitigate seizures but also influence mood disorders through modulation of serotonin and dopamine. Recent studies have uncovered their anticancer properties, demonstrated by both *in vitro* and *in vivo* experiments. This review comprehensively examines dibenzazepine’s pharmacodynamics, pharmacokinetics, and clinical applications, focusing on their emerging role in oncology. By highlighting the anticancer mechanisms of action—including apoptosis induction, inhibition of HDAC, Wnt/β-Catenin signaling, and Voltage-gated sodium channels, we suggest further research to fully elucidate their therapeutic potential and application in cancer treatment.

## 1 Introduction

Cancer remains one of the leading causes of mortality worldwide despite advances in conventional treatments such as surgery, chemotherapy, and radiation therapy. These standard modalities often have significant limitations, including drug resistance, off-target effects, and severe side effects, contributing to high mortality rates and poor quality of life for many patients (Yang et al., 2020; Tuck et al., 2023; Wills et al., 2024; Zolotykh et al., 2024). The development of drug resistance is one of the most intense challenges facing cancer treatment (Aroosa et al., 2023). Reasons for developing resistance include mutations in the drug target, reactivation of upstream or downstream signaling pathways, microenvironment-mediated resistance, intra-tumoral heterogeneity, and drug sensitivity (Ramos and Bentires-Alj, 2015). The high mortality rate, alongside increasing numbers of cases in recent years, highlights the importance of identifying novel therapeutic strategies that can overcome these barriers. Developing new anticancer drugs is complex, costly, and time-consuming, often requiring over a decade and billions of dollars before a new compound reaches clinical use (Juárez-López and Schcolnik-Cabrera, 2021). Drug repurposing—using existing drugs with established safety profiles for new therapeutic purposes—has emerged as a promising strategy to accelerate the availability of effective therapies. This approach allows researchers to bypass many early stages of drug development, reducing both time and cost (Lyne and Yamini, 2021; Malik et al., 2023). Thalidomide is a successful example of a repurposed candidate in cancer, initially designed for morning sickness in pregnancy, then got FDA approval for multiple myeloma (Gao et al., 2020). We have previously reported a few drugs with the potential for use in oncology, such as benzimidazoles, tetracyclines, and amino acetonitriles (Pourgholami et al., 2006; Pourgholami et al., 2013; Bahrami et al., 2021).

Dibenzazepine carboxamides, including carbamazepine (CBZ), oxcarbazepine (OXC), and eslicarbazepine (ESL), are well-known antiepileptic agents traditionally used to block voltage-gated sodium channels (VGSCs). In addition to their anticonvulsant properties, they are also effective in treating mood disorders, such as depression and bipolar disorder, by modulating neurotransmitter systems (Brodie, 2017; Lawthom et al., 2018; Maan et al., 2024). Recent studies have intriguingly revealed that dibenzazepine carboxamides possess potential anticancer properties, supported by both *in vitro* and *in vivo* evidence (Meng et al., 2011; Leslie et al., 2020; Zhao et al., 2020). Recent studies have shown that CBZ does not increase the risk of cancer in patients with epilepsy and even reduces it (Stritzelberger et al., 2021). For instance, our recent study demonstrated that eslicarbazepine induces apoptosis and cell cycle arrest in C6 glioma cells and significantly suppresses tumor growth in an intracranial rat model, underscoring its potential as an anticancer agent (Afsordeh et al., 2024). Furthermore, OXC and its metabolites have shown potent cytotoxic and genotoxic effects on human lymphocytes in culture media (Atlı Şekeroğlu et al., 2017).

This review aims to provide a comprehensive overview of dibenzazepine carboxamides’ pharmacodynamics, pharmacokinetics, and clinical applications, focusing on their emerging role in oncology. We will discuss the main molecular mechanisms underlying their anticancer activity, including apoptosis induction, deacetylases (HDACs) inhibition, suppression of Wnt/β-catenin signaling, and blockade of VGSCs. By highlighting these novel mechanisms, we advocate for further research to fully elucidate their therapeutic potential in cancer treatment and encourage their clinical evaluation as effective, repurposed anticancer agents.

## 2 History and structure

**FIGURE 1:**
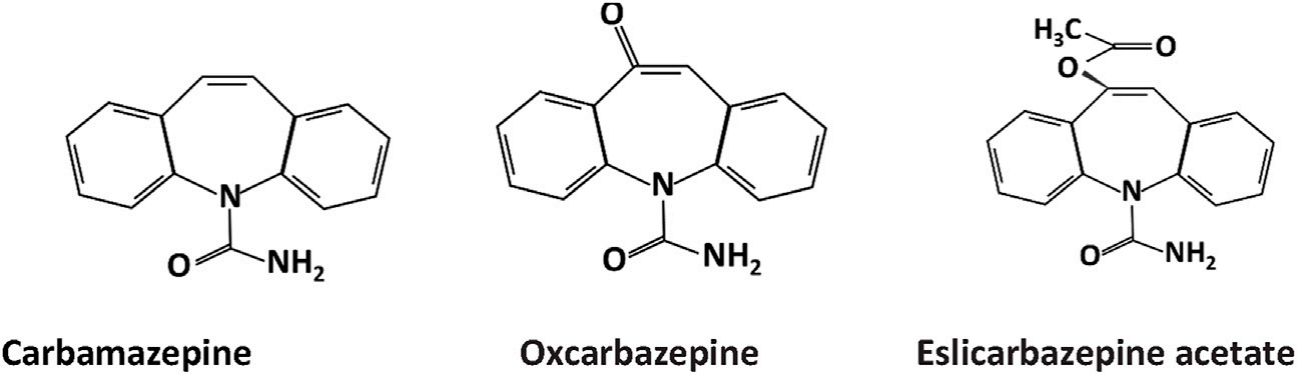
Chemical structure of dibenzazepine carboxamides .CBZ, OXC, and ESL all have a dibenzazepine nucleus bearing a 5-carboxamide substitute.

The primary chemical structure of the dibenzazepines carboxamides family is composed of a dibenzazepine with a 5-carboxamide nucleus, as depicted in Figure 1 (Aledo-Serrano and Gil-Nagel, 2020). The first drug of the family, carbamazepine [CBZ, (5-carbamoyl-5H-dibenzo (b,f) azepine)], was synthesized by Walter Schindler a Swiss chemist in 1953 (Benes et al., 1999). Its structure is quite similar to tricyclic antidepressants such as imipramine (Al-Waili, 2000). CBZ was initially FDA-approved for trigeminal neuralgia in 1968 (Blom, 1962). Showing anticonvulsant properties in the early 1960s led to its approval for epilepsy in Switzerland and Great Britain in 1963 and later FDA-approved for partial or tonic-clonic seizures in 1974 (Bonduelle et al., 1964; Shorvon, 2009). After being in clinical use for several years, CBZ has shown some adverse effects, including hyponatremia, responsible for the commonly reported CBZ-induced adverse drug reactions (nausea, headache, and dizziness), diplopia, and skin rashes (Fricke-Galindo et al., 2018). Due to these adverse effects of carbamazepine (Al Khalili et al., 2023), OXC (OXC, 10,11 -dihydro- 10-0x0-carbamazepine) was developed, which is the 10 keto analog of CBZ (Shorvon, 2000). It is a lipophilic compound with low water solubility and the same mechanism of action as CBZ but with less severe side effects (Mazza et al., 2007; Garoufi et al., 2016). The drug received FDA approval for partial epilepsy treatment in 2000 (Preboth, 2000). However, OXC has a relatively short half-life and limited formulation options (Galiana et al., 2017). Following these revelations, esclicarbazepine [ESL [(S)-(--)-10-acetoxy-10,11-dihydro-5H-dibenz[b,f]azepine-5-carboxamide] was developed. ESL is the newest drug and third-generation of dibenzazepines, with FDA approval for the treatment of epilepsy granted in 2013 (Tambucci et al., 2016). ESL has a structural distinction from CBZ and OXC, which lies in the hydroxyl group in the 10, 11-position of the central dibenzoazepine ring instead of a keto group. This structural change resulted in distinct metabolism, pharmacokinetics, and pharmacodynamic advantages (Galiana et al., 2017). ESL does not metabolize to carbamazepine-10, 11-epoxide, the culprit of CBZ-induced adverse effects (Galiana et al., 2017). ESL has shown high efficacy and safety when used for epilepsy management (Almeida and Soares-da-Silva, 2007).

## 3 Pharmacokinetics and pharmacodynamics

CBZ is predominantly metabolized in the liver, with less than 5% remaining unchanged (Kim et al., 2005). Cytochrome P450 subtype (CYP3A4) converts CBZ into its primary metabolite, carbamazepine-10,11-epoxide, which causes neurotoxicity (Aledo-Serrano and Gil-Nagel, 2020) and has anti-seizure effects like CBZ in animals (Bertilsson, 1978). About 15% of CBZ is metabolized to CBZ N-glucuronide by uridine diphosphate glucuronosyltransferase 2B7 (UGT2B7) (Fricke-Galindo et al., 2018). Another enzyme, myeloperoxidase (MPO), produces 2-hydroxycarbamazepine (2OH-CBZ), 3-hydroxycarbamazepine (3OH-CBZ), and 2,3 dihydroxycarbamazepine (Lu and Uetrecht, 2008). Approximately 75% of the drug is bound to plasma proteins (Bertilsson, 1978). After a single oral dose, the average half-life is 35 h, which decreases to 10–20 h after chronic use, leading to enzyme induction (Bertilsson, 1978). The maximum plasma concentration (C_max_) is reached or appears in 2–8 h (Spina et al., 1996), and the remainder of CBZ is excreted through urine (Bertilsson, 1978). CBZ passes through the placenta, and its plasma level decreases due to its high metabolism, making a relatively short plasma half-life in infants during the fetal period (Bertilsson, 1978). The most common adverse effects of CBZ use are dizziness, nausea, drowsiness, and weight gain (Koliqi et al., 2015). The side effects of this drug are usually mild in low doses, with severe side effects observed in high doses. Most patients treated with CBZ experience a decrease in the white blood cell count (Pellock, 1987; Delcker et al., 1997). In addition, the rate of change in the dose of CBZ can lead to psychomotor symptoms in the central nervous system (CNS), followed by gastrointestinal, hepatic, endocrine, and teratogenic side effects (Delcker et al., 1997; Tecoma, 1999). The second-generation carboxamide, OXC, is also metabolized by cytochrome P450 (CYP), CYP3A4, and CYP3A5 enzymes to 10, 11-dihydro-10-hydroxy-carbazepine (mono hydroxy derivative, MHD) in the liver (Schütz et al., 1986; May et al., 2003). After a single oral dose of OXC, peak concentrations (the highest level of medication in the blood) reach within approximately 1–3 h (May et al., 2003). The main route for OXC elimination is through renal excursion. The half-life of OXC has been reported to be between 1 and 5 h, whereas the half-life of MHD is up to 20 h. The MHD plasma protein binding rate is almost 40% (May et al., 2003). As the epoxide derived from CBZ is responsible for some of its toxic effects, the absence of this metabolite in OXC reduces the side effects (Shorvon, 2000). Studies have shown that symptoms such as nausea, digestive disorders, and ataxia may also occur with the use of OXC; however, the profile of side effects associated with the use of this drug is better than that of CBZ (Shorvon, 2000). OXC crosses the placenta in humans (May et al., 2003) and is not recommended for use during pregnancy (Shorvon, 2000). Owing to the structural change at positions 10 and 11, the third-generation drug, ESL, has a different metabolism. In the liver, enzyme CYP3A4 converts it into (S)-licarbazepine, oxcarbazepine (OXC), and (R)-licarbazepine, with the first enantiomer responsible for the most anticonvulsant activity of the drug through blockage of VGSCs (Almeida and Soares-da-Silva, 2007). The systemic amount of the ESL metabolite (eslicarbazepine) in the body after oral administration of ESL is 94% of the original dose (Bialer and Soares-da-Silva, 2012). ESL is more effective and has fewer side effects than the two earlier drugs, with the most adverse effects being non-serious (Ben-Menachem, 2010; Peltola et al., 2015; Guedes et al., 2023). Moreover, it also has additional benefits, including complete absorption after oral intake. Cytochrome P450 enzyme induction is much lower by ESL, and drug interactions are less prevalent (Shorvon, 2000). The drug decomposes with a half-life of 20–24 h (Almeida and Soares-da-Silva, 2007), and its binding rate to plasma proteins is less than 40% (Tambucci et al., 2016). ESL is also secreted 90% in urine (Almeida et al., 2009). Pharmacodynamic studies have shown that CBZ and OXC inactivate fast-voltage sodium channels, while ESL inactivates slow-voltage sodium channels (Lawthom et al., 2018). The pharmacokinetic and pharmacodynamic characteristics of the three drugs are compared in Table 1.

**TABLE 1:** Pharmacokinetics and Pharmacodynamics of dibenzazepines

	Site of metabolism	Metabolites	Enzymes involved in metabolism	Half-life	Excretion	Protein binding	Pharmacodynamic
CBZ	liver	Major: carbamazepine 10,11-epoxide (Pearce et al., 2008) Other metabolites include: 2-hydroxycarbamazepine (2OH-CBZ), 3-hydroxycarbamazepine (3OH-CBZ), 2,3 dihydroxycarbamazepine,(Pearce et al., 2008)	Cytochrome P450 (Pearce et al., 2002) Uridine diphosphate glucuronosyltransferase (UGT2B7) (Staines et al., 2004), Myeloperoxidase (Lu and Uetrecht, 2008)	35 h (range 18–65 h) (Bertilsson, 1978)	Urine (Bertilsson, 1978)	about 75% (Bertilsson, 1978)	inactivation of fast-voltage sodium channels
OXC	liver	10-hydroxycarbazepine (MHD) (licarbazepine) (Schütz et al., 1986)	P450 (CYP) CYP3A4, CYP3A5 (May et al., 2003)	1–5 h for oxcarbazepine and 7–20 h for MHD (May et al., 2003)	Urine (May et al., 2003)	50%–70%	inactivation of fast-voltage sodium channels
ESL	liver	Major: S-licarbazepine Oxcarbazepine (OXC) (R)-licarbazepine (Almeida and Soares-da-Silva, 2007)	CYP3A4	20–24 h (Almeida and Soares-da-Silva, 2007)	urine and fecal (Fotherby, 1995)	low (<40%) (Tambucci et al., 2016)	inactivation of slow-voltage sodium channels

## 4 Mechanism of action

The primary actions of dibenzazepines include anticonvulsant properties via inactivation of VGSCs (Aledo-Serrano and Gil-Nagel, 2020). The effects of dibenzazepines, especially CBZ, on neurotransmitters such as GABA, glutamate, dopamine, and serotonin have also led to their use in the treatment of mood disorders (Wang, 2013; Shah et al., 2015; Ayano, 2016). CBZ also inhibits calcium and potassium channels (Grunze et al., 1998). The following sections will discuss the common uses of dibenzazepines in detail based on their mechanism of action.

## 5 Clinical evidence

### 5.1 Epilepsy

**FIGURE 2:**
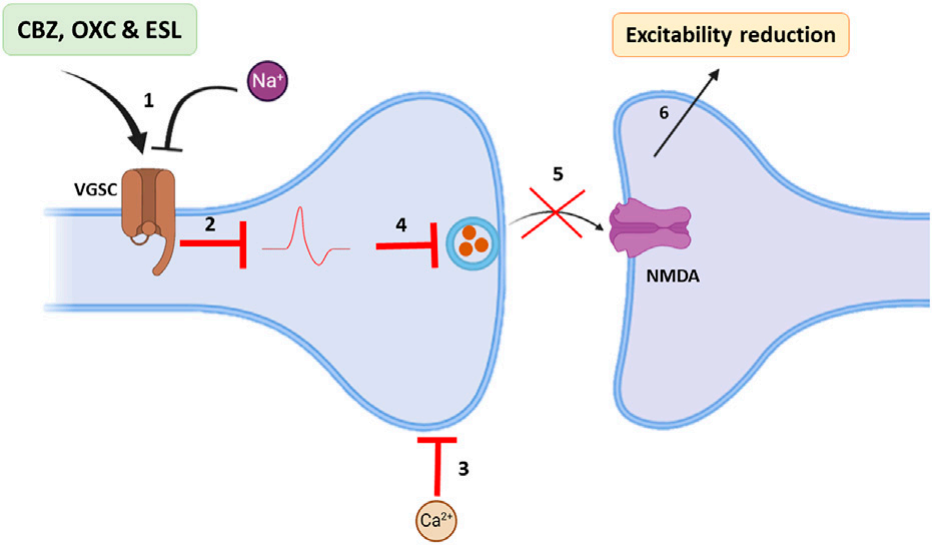
Main antiepileptic actions of dibenzazepines. 1: VGSCs of brain neurons become impermeable to sodium in the presence of dibenzazepines; 2: Hence, action potentials are blocked. 3: In addition, calcium entrance from synaptic space decreases. 4 and 5: Therefore, glutamate release and its effects on NMDA receptors are declined. 6: All these events reduce brain excitability. Voltage-gated Na+ channels: VGSCs, N-methyl-D-aspartate: NMDA. By Biorender.com.

Based on Na channel blocking abilities, dibenzazepines have anticonvulsant properties. Epilepsy is one of the most common diseases of the CNS that occurs with simultaneous abnormal activity in the function of brain neurons (Hu et al., 2023). With an approximate prevalence of 6 per 1,000 people (Brigo et al., 2022), it is one of the common public health challenges for physicians worldwide. More than 30 different drugs have been used to manage epilepsy (Hu et al., 2023). These drugs can improve the acute conditions of patients by influencing multiple molecular mechanisms (Rubio et al., 2023); among them, dibenzazepines, including CBZ, OXC, and ESL, are sodium channel blockers that treat epilepsy alone or in combination with other drugs (García-Peñas et al., 2020; Rocamora et al., 2020; Watkins et al., 2020; Nevitt et al., 2022). Through binding to the inactive state of the sodium channels in the membrane, CBZ slows down their reactivation. This delay in sodium channel reactivation reduces the chance of rhythmic propagation of action potentials and prevents the calcium entrance into synaptic membranes. Finally, synaptic function decreases with CBZ treatment, which reduces the excitability of neurons in the brain by increasing the release of GABA and decreasing glutamate release (Tolou-Ghamari et al., 2013). Figure 2 illustrates the anticonvulsant mechanism of dibenzazepines. The antiepileptic effects of OXC are the same as CBZ (Faught and Kim, 2015). The anticonvulsant effects of CBA and OXC have been demonstrated in animal studies of spontaneous seizures induced by kainite (Grabenstatter et al., 2007), pentylenetetrazol (PTZ) (Barzroodi Pour et al., 2021), and maximal electroshock (Tecoma, 1999; Zhen Sun et al., 2002). Double-blind studies have shown that oxcarbazepine use is associated with fewer side effects and better tolerance (Reinikainen et al., 1987). With the entry of the third-generation drug of this family (ESL) into the clinic, better seizure control and fewer side effects led to improved conditions for seizure control in epileptic patients (Mestre and Ferreira, 2009; Verrotti et al., 2014; Soares-da-Silva et al., 2015).

### 5.2 Mood stabilization

It was later found that dibenzazepine carboxamides have other clinical uses, including mood stabilization (Maan et al., 2023). CBZ is used to treat bipolar disorder (Hirschfeld and Kasper, 2004), manic-depression (Ballenger and Post, 1980), attention-deficit hyperactivity disorder (ADHD) (Silva et al., 1996), and neuropathic pain (Buescher, 2006; Wiffen et al., 2014). OXC also showed promising effects on bipolar disorders in clinical studies (Centorrino et al., 2003; Benedetti et al., 2004). In patients with bipolar disorder, the level of GABA neurotransmitter decreases, which causes excitotoxicity and cell death through apoptosis. Chronic administration of CBZ as an agonist of GABA receptors α1, β2, and γ2 subunits can stabilize mood in these patients (Ayano, 2016). The mood-modifying effects of CBZ occur through five of the neurotransmitters in the brain, including A) Glutamate: CBZ has anti-glutamatergic effects by reducing its release and inhibiting calcium influx, which reduces the post-synaptic effects of glutamate (Kawata et al., 2001). B) Dopamine: dopamine is involved in the pathophysiology of bipolar disorder. CBZ indirectly affects dopaminergic receptors through the D2 receptor phosphorylation and density reduction (Kawata et al., 2001). C) Serotonin: like dopamine, serotonin affects the pathophysiology of bipolar disorder. CBZ controls this disease by increasing the release of serotonin and inhibiting its reabsorption (Dailey et al., 1998; Kawata et al., 2001). D) Norepinephrine: CBZ also reduces the level of norepinephrine, the most crucial neurotransmitter in bipolar disorder (Norman et al., 2009). However, the mood-stabilizing properties of CBZ are a matter of question as it has been shown that CBZ is a selective adrenaline A1 receptor antagonist and A2A receptor agonist (Van Calker et al., 1991; Okada et al., 2019). Activation of A2A receptors is attributed to depression-like symptoms, while increased A1 receptor signaling is associated with antidepressant effects (van Calker et al., 2019). Through antagonistic action on the A2A receptor, chronic administration of CBZ suppresses the activation of astroglial glutamatergic transmission induced by proinflammatory cytokines Interferon-gamma (IFNγ) and tumor necrosis factor-alpha (TNFα), implying the benefits of CBZ in the prevention of pathomechanisms development in several neuropsychiatric disorders, such as Niemann–Pick disease, schizophrenia, and autism (Okada et al., 2019). Other mechanisms include blocking cyclic adenosine monophosphate and G protein and increasing brain-derived neurotrophic factor (BDNF) (Ambrósio et al., 2002; Sadock et al., 2009; Ayano, 2016).

**FIGURE 3:**
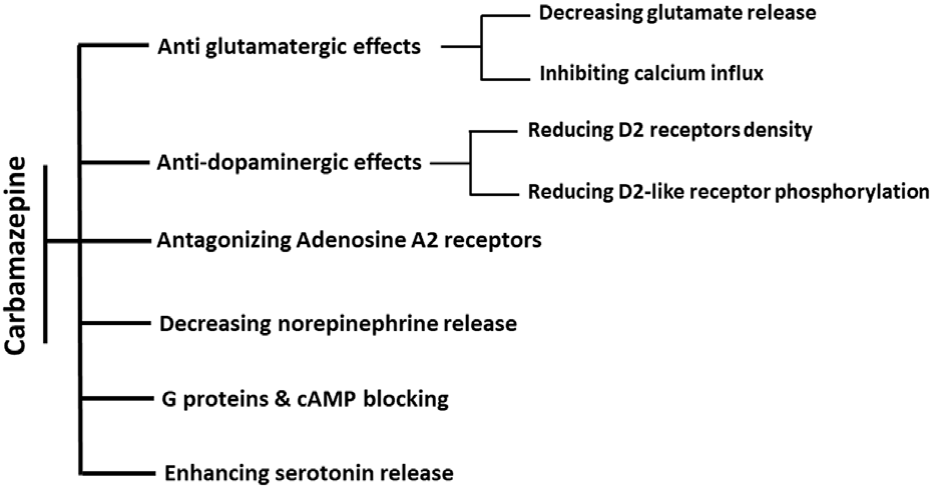
Summary of known actions of dibenzazepines in Mood stabilization. The effects of CBZ on mood regulation are through six mechanisms. Antiglutamatergic effects are through reducing glutamate release and calcium influx inhibition. The decrease in dopamine efficacy is induced by the decline in both the density and phosphorylation of D2 receptors. Moreover, these effects are shown by increasing the release of serotonin, decreasing the release of norepinephrine, antagonistic effects of adenosine on A2 receptors, and blocking the effects of cAMP and G proteins.

Another member of the family, OXC, has also been suggested as a potential treatment for ADHD in adults (Davids et al., 2006). Similar effects from OXC have been observed in controlling impulsive aggression in adults (Mattes, 2005). In this context, other studies have also suggested and demonstrated the mood-stabilizing effects of ESL (Popova et al., 2007; Nath et al., 2012). Moreover, its efficacy and safety in trigeminal neuralgia have been established (Sanchez-Larsen et al., 2018; Fortuna, 2022). Altogether, these data indicate the efficiency of carbamazepine and its derivatives in controlling mood disorders. Figure 3 shows the mood stabilization activities of dibenzazepines.

## 6 Anticancer effects of dibenzazepines

In addition to the aforementioned neurotherapeutic effects, these agents may also have anticancer properties. Over the past decades, some studies have provided encouraging results, suggesting that the pharmacological properties of dibenzazepines may interfere with cancerous tumor growth.

Dibenzazepines exert their anticancer effects through a variety of molecular mechanisms. These mechanisms include the induction of apoptosis and modulation of the cell cycle, autophagy induction, histone deacetylases (HDACs) inhibition, voltage-gated Na+ channels (VGSCs) inhibition, and interference with Wnt/β-catenin signaling. Dibenzazepines exhibit a multifaceted approach to cancer treatment by targeting multiple pathways, setting the stage for their potential use as anticancer agents. Understanding these pathways provides crucial insight into how these drugs may be repurposed for cancer therapy. The following section will explore the existing evidence for the anticancer effects of dibenzazepines, highlighting the leading studies demonstrating their efficacy in various cancer models.

### 6.1 Apoptosis induction

Preliminary studies have shown the activation of apoptosis signaling in response to dibenzazepine exposure (Ota et al., 2021). Activation of apoptosis in platelets induces thrombocytopenia and bleeding in patients treated with CBZ. These pathways include decreased phosphorylation of the Bcl-xl/bcl-2-associated death promoter (BAD), vasodilator-stimulated-associated phosphoprotein (VASP), and glycoprotein 1b-beta (GPIbβ) in platelets, indicating an inhibitory effect on protein kinase A (PKA). In addition, PKA activity is reduced through PI3K/Akt/PDE3A signaling, resulting in apoptosis in platelets (Xiao et al., 2021). Through the activation of the Ras/Raf/ERK/p53 signaling pathway, CBZ induces hepatic DNA damage and mitochondrial apoptosis in Chinese rare minnows (Yan et al., 2021). After 60 days of daily oral CBZ intake (25 mg/kg) by Wistar-Albino rats, Caspase-3, PARP-1, and 8-hydroxy-2-deoxyguanosine immunoreactivity markedly increased, indicating apoptosis and oxidative stress as culprits of induced renal toxicity by CBZ (Erdem Guzel et al., 2023).

Administering CBZ and OXC on days 5–17 of pregnancy in rats leads to pro-apoptotic effects in the CA1, CA3, and dentate gyrus (DG) regions in the hippocampus of the offspring (González-Maciel et al., 2020). OXC-induced apoptosis occurs in the brain cells of adult and neonatal rats due to the activation of caspase-3 and Bax/Bcl-2 signaling (Song et al., 2018). Flow cytometric analysis showed that OXC caused the mitosis-phase cell cycle arrest and increased histone H3 phosphorylation, indicating cell mitosis. Also, it inhibited centrosome separation by reducing Polo-like kinase 1 (PLK1) activation and inducing apoptosis in NRK-52E cells via abnormal spindle formation (Ota et al., 2021). Cancer studies have demonstrated the induction of apoptosis by dibenzazepines. A recent *in vitro* study using MTT assay and AO/EB staining showed that CBZ induces apoptosis and cytotoxicity in the HT-29 human colon adenocarcinoma cell line (Sohaib and Ezhilarasan, 2020). In addition, immunofluorescence analysis showed that CBZ also increased caspase-3 activity, one of the key mediators in intrinsic apoptosis (Sohaib and Ezhilarasan, 2020). CBZ has protective and mitigating effects against ionizing irradiation in Murine Cells (Kim et al., 2012). Clinical observations using a wide range of anticonvulsants showed that among anticonvulsant drugs, CBZ significantly reduces the risk of skin and digestive cancers (Stritzelberger et al., 2021). OXC has anti-proliferative activity in HeLa and MCF7 cell lines and, to a lesser extent, in HepG2 (El Sharkawi et al., 2014). In human glioma cell lines with isocitrate dehydrogenase (IDH) mutations, OXC induces apoptosis with increased caspase 3/7 activity. OXC also inhibits cell growth and proliferation effects *in vitro* in glioma stem-like cells (Dao Trong et al., 2023). Recent studies have highlighted the potential anticancer effects of ESL, particularly in glioma models. ESL has been shown to induce apoptosis and promote G2/M cell cycle arrest in C6 glioma cells, suggesting it may be a promising candidate for drug repurposing in glioma treatment (Afsordeh et al., 2024).

These studies suggest a robust potential for dibenzoazepine carboxamides to activate apoptosis and exert anticancer effects. However, more precise studies, including well-designed preclinical and clinical trials, are needed to fully elucidate their therapeutic potential and optimize their use in oncology.

### 6.2 Autophagy

Autophagy is a cellular process in which damaged and abnormal cellular materials are removed by lysosomes. Autophagy has protective roles against many diseases (Debnath et al., 2023). However, the mechanism of autophagy in cancer treatment is controversial, and interventions to stimulate or inhibit autophagy in various cancers are proposed according to their underlying cause (Levy et al., 2017). HDAC inhibitor drugs such as CBZ can induce apoptosis and autophagy by inhibiting the PI3K-Akt-mTOR pathway (Aroosa et al., 2023). CBZ induces autophagy in liver cells by activating calpain, reducing Atg7 and Beclin-1 (Kim et al., 2013). SMARCA4 is a BRG1 protein-encoding gene identified as an oncogene in many cancers (Schiebler et al., 2015; Zhang et al., 2018). Recently, Shaykevich et al. have reported that CBZ lowers BRG1 protein and mRNA levels in KRAS-mutant colorectal cancer cells and upregulates it in KRAS-wild type ones. They also sought the ULK1 mRNA level (an initiator of autophagy) and found that CBZ decreased the ULK1 mRNA KRAS-mutated colorectal cancer cells at both 6 and 24 h. These results show that CBZ can inhibit ULK1 in KRAS-mutated cells, potentially leading to a reduction in autophagy and a subsequent decrease in SMARCA4 levels (Shaykevich et al., 2024). Also, at different concentrations, CBZ protected murine hematopoietic progenitor 32D cl 3 cells against radiation and induction of autophagy (Kim et al., 2011). Another study reported the protective effects of CBZ on radiation independent of autophagy (Kim et al., 2012). Although limited, based on these observations, this drug class seems to be an inducer of autophagy in some cells and conditions. This autophagy induction inevitably suggests an intriguing path for further research in this field and the autophagy-mediated treatment of various types of cancer.

### 6.3 HDAC inhibition

Histone deacetylases are enzymes that remove acetyl groups from lysine residues of both histone and nonhistone proteins, leading to chromatin condensation and repression of gene expression (Seto and Yoshida, 2014), by which they affect a myriad of biological events such as cell cycle, differentiation, and apoptosis in cancer cells (Marks et al., 2000; Zhang and Zhong, 2014). There are three main groups of histone deacetylases: class I HDACs (1, 2, 3, and 8) are homologous to the yeast Rpd3 gene, ubiquitously expressed, and primarily located in the nucleus. Class II HDACs (4–10, except 8) are gene products that actively shuttle between the nucleus and the cytosol. Class III HDACs, such as Sir2, are protein deacetylases that appear to have targets other than histones (Marks et al., 2003). HDAC dysregulation contributes to tumor development and progression, and HDAC inhibitors are considered promising anticancer agents (Liang et al., 2023). Through epigenetic regulation of gene expression, HDAC inhibitors are crucial in inducing cancer cell cycle arrest, promoting differentiation, and triggering cell death. They also reduce angiogenesis and modulate the immune response (Eckschlager et al., 2017). Moreover, several laboratory and clinical studies have demonstrated the effectiveness of CBZ in inhibiting histone deacetylases in a cancer context. CBZ and its major metabolite CBZ-10, 11-epoxide inhibit HDAC (3 and 7) in the HepG2 liver carcinoma cell line (Beutler et al., 2005). CBZ causes degradation of the Her2 protein through proteosomes, and these effects are related to inhibiting HDAC in breast cancer (Meng et al., 2011). CBZ inhibits histone deacetylase type 6 in breast cancer by increasing alpha-tubulin acetylation (Beutler et al., 2005). Two of the most-known effects of CBZ, including mood stabilization and teratogenicity, are caused by histone deacetylase inhibitory (HDACi) properties (Phiel et al., 2001; Beutler et al., 2005).

Furthermore, it has been shown that CBZ decreases interleukin-6 and prostate-specific antigen (PSA) secretion in different human prostate cancer cell lines (Abdul and Hoosein, 2001). Treatment of the LNCaP cell line (prostate cancer) with CBZ and OXC reduces PSA levels. Also, chronic exposure to CBZ and OXC slows the transformation of pre-tumor cells into prostate cancer and reduces its risk (Stettner et al., 2012). In line with the preventive effects, a case-control study found that CBZ reduced the risk of advanced prostate cancer (Salminen et al., 2016). In addition, a new study in 2021 showed that CBZ users had a lower risk of advanced prostate cancer than other HDAC-inhibiting antiepileptic drugs, such as valproate and phenobarbital (Salminen et al., 2021). However, regarding the prognosis of this disease with dibenzazepines, Salminen et al. have reported no beneficial effect on the mortality caused by this cancer in the users of HDAC inhibitor antiepileptic drugs compared to other antiepileptic drugs (Salminen et al., 2022). These findings indicate that dibenzazepines can modulate cancer progression through HDAC inhibition.

### 6.4 VGSC inhibition

Dibenzazepines primarily act through the VGSCs inhibition (Hebeisen et al., 2015). VGSCs are composed of nine α (Nav1.1-Nav1.9) and four β subunits (β1-4) (Goldin et al., 2000; Brackenbury and Djamgoz, 2006). Researchers have shown that these sodium channel functions are phagocytosis, endocytosis, secretion, proliferation, and differentiation in non-excitable cells (Xia et al., 2017). For example, in human colon cancer, the SCN5A gene, which encodes Nav1.5 isoform, plays a critical role in cell invasion (House et al., 2010). VGSCs are aberrantly expressed in multiple human cancers (Bian et al., 2023). Compared to normal brain tissue, Nav1.2 and Nav1.6 subunits are highly expressed in glioma tissue (Griffin et al., 2020). The protein expression of Nav1.3 and Nav1.6 subunits increases in low-grade astrocytoma (Guan et al., 2018). In addition, a study has reported the role of the Nav1.7 subunit in gastric cancer (Xia et al., 2016). In non-small cell lung cancer, this invasive pathway occurs in the Nav1.7 subunit through the epidermal growth factor (EGF) - signal-regulated and kinase 1/2 (ERK1/2) pathway (Campbell et al., 2013). Also, in human cervical cancer, overexpression of Nav1.5 has been implicated in cell invasion (Hernandez-Plata et al., 2012). A recent meta-analysis study confirmed that VGSCs promote prostate cancer cell metastasis and suggested targeting VGSCs as a treatment option (Yildirim-Kahriman, 2023). Recently, studies by Theresa et al., in 2020 showed that ESL inhibited both transient and persistent sodium currents, demonstrating, for the first time, the potent inhibitory effects of ESL on the Nav1.5 isoform. Many cancer cells express Nav1.5 and voltage-dependent sodium channels that enhance invasion and metastasis, and inhibiting these channels may be effective in cancer control (Leslie et al., 2020). In addition, another mechanism that can affect the metastasis and migration of cancer cells under the influence of these drugs is the process of inhibiting nuclear factor kappa B (NF-κB) activity CBZ, also significantly inactivates the motility proteins, including myosin light chain 2 (MLC2), myosin phosphatase 1 target subunit 1 (MYPT1), and focal adhesion kinase (FAK), which are necessary for lymph endothelial cell migration and can reduce metastasis in a three-dimensional model of breast cancer cell culture (Teichmann et al., 2014).

Considering the role and importance of VGSCs and their subunits in the pathophysiology of all types of cancer, especially cell invasion and metastasis, it is necessary to identify the mechanisms behind these processes. Based on these observations and reports, dibenzazepines are worthy of further research in this area of oncology.

### 6.5 Wnt/β-Catenin signaling inhibition

Wnt (Wingless/Integrated) signaling is crucial in physiological processes such as embryonic development and adult homeostasis (Zhong and Virshup, 2020). Wnts are a broad family of glycoproteins that regulate multiple developmental pathways (Boyer et al., 2010; Grishanova et al., 2023). Wnt signaling occurs in two pathways. A) β-catenin dependent (canonical) or B) nondependent or non-canonical (Zhan et al., 2017). Dysregulation of this pathway is a hallmark of many cancers, where overactive Wnt signaling leads to increased β-catenin accumulation in the cytoplasm and its subsequent translocation to the nucleus. In the nucleus, β-catenin binds to T-cell factor/lymphoid enhancer factor (TCF/LEF) transcription factors, promoting the transcription of genes involved in cancer cell proliferation, invasion, and metastasis(Yu et al., 2020). Therefore, inhibiting this pathway can be one of the novel strategies to control many cancers (Chen et al., 2017; Yang et al., 2018; Rogaczewski et al., 2022). CBZ has been investigated as a Wnt/β-catenin signaling inhibitor in several studies (Parveen et al., 2018; Im et al., 2019; Bai et al., 2021).

**FIGURE 4:**
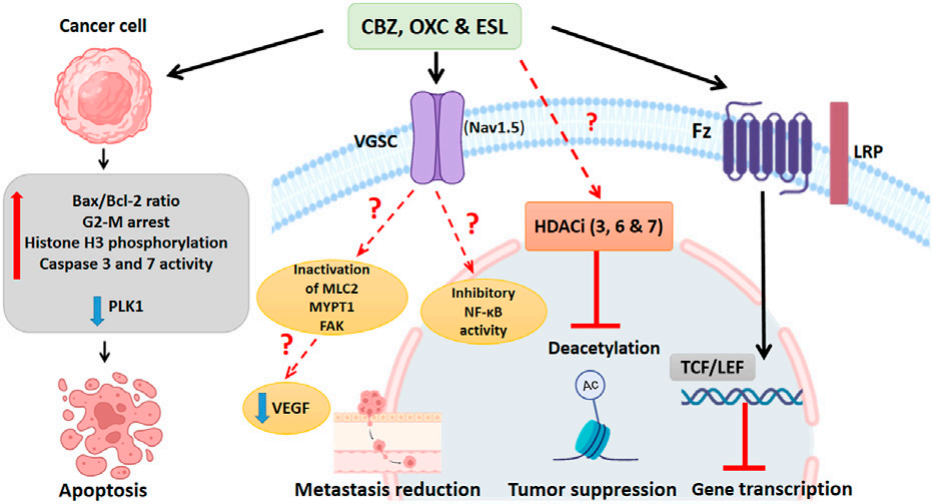
Summary of known actions of dibenzazepines in cancer. Cancer cells exposed to dibenzazepines increase the ratio of Bax/Bcl2 ratio and caspase 3 and 7, induce G2-M cell cycle arrest, enhance histone H3 phosphorylation, and decrease the activity of PLK1, all leading to apoptotic signaling. Dibenzazepine’s action through VGSCs with unknown mechanisms leads to a decrease in the NF-kB activity, MLC2, MYPT1, and FAK lead to a reduction in metastasis of cancer cells treated with these drugs. Also, dibenzazepines reduce VEGF levels. Dibenzazepines also inhibit the deacetylation of chromatin by inhibiting histone deacetylases (3, 6, and 7) and suppress tumor growth. They also inhibit the Wnt/β-catenin pathway by binding to the FZ receptor and finally inhibit gene transcription. NF-kB: Nuclear factor kappa-light-chain-enhancer of activated B cells, PLK1: Polo-like kinase 1, MLC2: myosin light chain 2, MYPT1: myosin phosphatase 1 target subunit 1, FAK: focal adhesion kinase, LRP: Lipoprotein receptor-related proteins, FZ:*N-terminal extra-cellular cysteine-rich domain of a Frizzled,* TCF/LEF: T cell factor/lymphoid enhancer factor. By Biorender.com.

*In vitro*, studies have shown the effectiveness of CBZ in human colon cancer cell lines (SW480) and suggested that this drug probably plays a role in reducing the levels of VEGF and β-catenin in this cell line (Akbarzadeh et al., 2016) and mouse adipocyte 3T3-L1 cells (Im et al., 2019). In addition, recently, surface plasmon resonance (SPR) studies showed that CBZ is a ligand for the FTZ receptor of Wnt/β-catenin signaling (Zhao et al., 2020), which specifically binds to a novel pocket on the Frizzled-8 (FZD8_CRD_), and does not overlap with the known Wnt binding sites, suggesting that CBZ may directly inhibit Wnt signaling by interfering with receptor-ligand interactions. FZD proteins, as essential Wnt receptors, are a central point for Wnt signaling intervention in diseases such as cancer (Zhong and Virshup, 2020). Figure 4. Shows the anticancer mechanisms of dibenzazepines.

## 7 Potential for the management of brain tumors

Seizures are well-known symptoms of early brain tumors and can occur at any point during the disease, considered a warning sign for the diagnosis or progression of brain tumors (Pignatti et al., 2002). The incidence of seizures is about 80% in patients with low-grade gliomas (LGGs) (Kurzwelly et al., 2010) and 60% in high-grade gliomas (HGGs) (van Breemen et al., 2007). Seizures in patients with LGGs are most resistant to drug treatments (Berntsson et al., 2018). The extensive use of antiepileptic drugs in brain tumors and their potential for cancer control made them highly desirable for both laboratory and clinical studies.

Patients with brain tumors and seizures who do not receive antiepileptic drugs have shorter survival rates (Cacho-Diaz et al., 2018). CBZ has similar effects to valproate in the survival of patients with GBM owing to its inhibitory effects on histone deacetylation (Guthrie and Eljamel, 2013). Carbamazepine, oxcarbazepine, and Eslicarbazepine are all FDA-approved drugs utilized clinically in epileptic seizures. This is a huge advantage to patients with GBM suffering from seizures.

In addition, *in vitro* tests on human glioma cell lines U-87 MG and T98G revealed the efficacy of OXC to be equal to temozolomide in MTT and cell cycle tests (Lee et al., 2016). CBZ also suppresses cell growth of A 172, AM 38, and YH 13 cell lines (Yagi et al., 2022). A cohort study showed the effectiveness of ESL on tumor-induced seizures in glioma patients (Zoccarato et al., 2021). The promising results of ESL in our recent findings of reduced tumor growth and induced apoptosis in glioma cells suggest that this drug warrants further investigation in clinical trials to assess its therapeutic potential in patients with brain tumors (Afsordeh et al., 2024). Table 2. Summarizes reported studies on dibenzazepines in cancer. As mentioned, one of the main symptoms associated with brain tumors is seizures. Due to the prominent role of dibenzazepines in controlling seizures and the positive results of preliminary studies in inhibiting the growth of brain cancer cell lines, dibenzazepines may be suitable candidates for further investigation to inhibit tumor growth in addition to seizure control. The promising outcomes from preclinical studies warrant further investigation into their mechanisms of action and therapeutic benefits in GBM as a priority.

**TABLE 2:** Summary of studies evaluating the effect of dibenzazepines in cancer.

Author/Year	Drug name	Model	Cancer type	Mechanisms of action/effect(s)
Beutler et al. (2005)	CBZ and their major metabolite CBZ-10,11-epoxide	*In vitro*	HepG2 liver carcinoma cell line	HDAC inhibition (3 and 7)
Meng et al. (2011)	CBZ	*In vitro*	SK-BR-3 and MDA-MB-231 cell lines	HDAC inhibition (6)
Stettner et al. (2012)	CBZ and OXC	*In vitro*	LNCaP cell line,prostate cancer	Reduced PSA expression in mRNA and protein levels
Stettner et al. (2012)	CBZ and OXC	Clinical	Prostate cancer	Reduced PSA of levels
Guthrie et al. (2013)	CBZ	Clinical	Glioblastoma	HDAC inhibition
El Sharkawi et al. (2014)	OXC	*In vitro*	HeLa, MCF7 and HepG2 cell lines	Anti-proliferative
Lee et al. (2016)	OXC	*In vitro*	U-87 MG and T98G cell lines Glioma	Inhibiting cell growth and arrest in the cell cycle
Akbarzadeh et al. (2016)	CBZ	*In vitro*	SW480 cell lines Human colon cancer	Reducing the level of β-Catenin
Akbarzadeh et al. (2016)	CBZ	*In vitro*	SW480 cell lines Human colon cancer	Reducing the level of VEGF
Zhao et al. (2020)	CBZ	*In vitro*	Surface plasmon resonance	suppresses as a specific ligand for FTZ receptor of Wnt/β-Catenin signaling
Leslie et al. (2020)	ESL	Electrophysiology	MDA-MB-231 cell line metastatic breast carcinoma	Inhibitory Nav1.5 isoform and Inhibitory invasion and metastasis
Sohaib and Ezhilarasan et al. (2020)	CBZ	*In vitro*	HT-29 cell line, Human Colon Adenocarcinoma	Apoptosis
Dao Trong et al. (2023)	OXC	*In vitro*	Human glioma IDH mutant cell	Apoptosis and anti-proliferative
Afsordeh et al. (2024)	ESL	*In vitro*	C6 cell line	Apoptosis and arrest in the cell cycle
Afsordeh et al. (2024)	ESL	*In vivo*	Animal model	Suppresses tumor growth

## 8 Valproic acid

Previous studies have shown that valproic acid, another antiepileptic drug, also has anti-cancer potential through tumor growth reduction and metastasis suppression (Blaheta and Cinatl, 2002). This drug, shares signaling pathway crosstalk with dibenzazepines. It inhibits WNT and histone deacetylases and has shown effectiveness in some *in vitro* and *in vivo* cancer studies(Duenas-Gonzalez et al., 2008). On the other hand, laboratory studies have shown that treatment with valproic acid, a histone deacetylase inhibitor, was beneficial in animals with diabetes-related colon cancer (Patel and Patel, 2018). This is while valproic acid, used to treat epilepsy have not shown a protective role against cancer (Hallas et al., 2009). Also, the use of valproic acid as a histone deacetylase inhibitor with a cumulative dose of 1,500 g over 5 years is not associated with a reduced risk of cancer (Blaheta et al., 2002). Given that valproic acid, like dibenzazepines, is a well-known drug in the treatment of epilepsy and bipolar disorders, and studies have shown that it can also be useful for controlling cancer, further studies *in vitro* and in systematic reviews are needed to compare these drugs to reach a definitive conclusion about their advantages and disadvantages. However, there is still much work to be done to understand the effectiveness of these drugs on cancer and reduce the suffering of patients.

## 9 Conclusion

Dibenzazepine carboxamides, first known as anticonvulsants and mood stabilizers, exhibit potential as multifunctional anticancer agents due to their ability to target multiple signaling pathways in cancer. This review has highlighted several of their promising mechanisms of action, including the induction of apoptosis, modulation of the cell cycle, and inhibition of key oncogenic pathways such as HDACs and Wnt/β-catenin signaling. However, the current body of evidence on the anticancer properties of dibenzazepines is limited, with most studies conducted at the preclinical level. The lack of clinical studies is a significant gap that needs to be marked to validate their therapeutic potential in oncology. Given their established safety profiles and known pharmacokinetics, repurposing dibenzazepines, particularly ESL, offers a strategic advantage by potentially bypassing the high costs and extended timelines associated with the new anticancer drug development. Future research should focus on further exploring these compounds in diverse cancer models, elucidating their precise mechanisms of action, optimizing dosing regimens, and advancing toward pilot clinical trials. There is a need for future studies to explore the pharmacokinetics and safety profiles of dibenzazepines in the context of cancer. Such efforts are essential a complete assessment of their anticancer capabilities. The transition from anticonvulsant use to cancer therapy exemplifies the innovative potential of drug repurposing and opens new horizons in the search for effective, multi-targeted cancer treatments.

## Abbreviations

CBZ, carbamazepine; OXC, oxcarbazepine; ESL, eslicarbazepine; VGSCs, voltage-gated Na^+^ channels; CYP3A4, Cytochrome P450 subtype; UGT2B7, uridine diphosphate glucuronosyltransferase; MPO, myeloperoxidase; 2OH-CBZ, 2-hydroxycarbamazepine; 3OH-CBZ, 3-hydroxycarbamazepine; CYP, cytochrome P450; PTZ, pentylenetetrazol; ADHD, attention-deficit hyperactivity disorder; BDNF: brain-derived neurotrophic factor; IFNγ, Interferon-gamma; TNFα, tumur necrosis factor-alpha; VASP, vasodilator-stimulated-associated phosphoprotein; GPIbβ, glycoprotein 1b-beta; PKA, protein kinase A; PLK1, Polo-like kinase 1; IDH, isocitrate dehydrogenase; HDAC, histone deacetylases; PSA, prostate-specific antigen; EGF, epidermal growth factor; MLC2, myosin light chain 2; MYPT1, myosin phosphatase 1 target subunit 1; FAK, focal adhesion kinase; SPR, surface plasmon resonance; FZD, Frizzled; HGGs, high-grade gliomas; LGGs, low-grade gliomas.
